# Transient Nephrotic Syndrome in an Infant With Heterozygous Variants of Uncertain Significance in LMX1B and TTC21B

**DOI:** 10.7759/cureus.111908

**Published:** 2026-07-01

**Authors:** Alison Greisch, Elise Hennaut, Khalid Ismaili

**Affiliations:** 1 Pediatrics, Centre Hospitalier Interrégional Edith Cavell, Brussels, BEL; 2 Pediatric Nephrology, Queen Fabiola Children's University Hospital, Brussels, BEL

**Keywords:** genetic nephropathy, infant, lmx1b, nephrotic syndrome, pediatric nephrology, transient nephrotic syndrome, ttc21b

## Abstract

Infantile nephrotic syndrome is rare and frequently associated with an underlying genetic cause. Oligogenic inheritance has been proposed in selected cases, although supporting evidence remains limited.

We report the case of an 8-month-old infant presenting with nephrotic syndrome characterized by massive proteinuria (25 g/g creatinine), hypoalbuminemia (17 g/L), and preserved renal function. Genetic testing using a targeted nephrotic syndrome panel identified two heterozygous variants of uncertain significance (American College of Medical Genetics and Genomics (ACMG) class 3) in *LMX1B *and *TTC21B*. The patient's mother carried both variants, whereas the maternal grandfather carried only the *LMX1B* variant. No corticosteroids or immunosuppressive therapy were administered. Supportive treatment alone resulted in complete remission by 12 months of age, which has been maintained through the latest follow-up at 3 years and 8 months.

The coexistence of two variants of uncertain significance raises the possibility of a genetic contribution to the phenotype; however, the available evidence is insufficient to establish a causal oligogenic mechanism. This case highlights the unusual occurrence of sustained spontaneous remission in infantile nephrotic syndrome and illustrates the challenges of interpreting variants of uncertain significance in clinical practice.

## Introduction

Nephrotic syndrome (NS) is one of the most common glomerular diseases in children, with an estimated incidence ranging from 1.15 to 16.9 cases per 100,000 children. It is characterized by massive proteinuria, hypoalbuminemia, edema, and hyperlipidemia. Most cases occurring after two years of age are idiopathic and responsive to corticosteroid therapy [1,2]. In contrast, a genetic etiology is identified in approximately 70% of NS cases presenting during the first year of life.

Recent advances in molecular genetics have led to the identification of pathogenic variants in more than 30 genes involved in podocyte structure and function, the glomerular basement membrane, or ciliary function [3,4]. Among these genes are *LMX1B*, inherited in an autosomal dominant manner, and *TTC21B*, typically associated with an autosomal recessive disease.

The *LMX1B* gene, located on chromosome 9, encodes a LIM-homeodomain transcription factor essential for limb, glomerular basement membrane, and podocyte development. Pathogenic variants in *LMX1B* are classically associated with nail-patella syndrome (also known as osteo-onychodysplasia), characterized by nail dysplasia, patellar hypoplasia or aplasia, elbow abnormalities, and renal involvement [5]. However, isolated renal manifestations without extrarenal features have also been described [6].

The *TTC21B* gene encodes intraflagellar transport protein 139 (IFT139), a key component of retrograde transport within the primary cilium of renal tubular cells [7]. Biallelic pathogenic variants in *TTC21B* have been associated with ciliopathies such as nephronophthisis, Jeune syndrome, Joubert syndrome, and some forms of focal segmental glomerulosclerosis. In heterozygous individuals, *TTC21B* variants may act as genetic modifiers contributing to the severity or variability of pre-existing kidney disease [8].

Although oligogenic inheritance has been proposed in some forms of nephrotic syndrome, evidence remains limited, and the contribution of multiple variants to disease expression is often difficult to establish. Interpretation is particularly challenging when variants are classified as variants of uncertain significance and segregation data are inconclusive [9].

We report the case of an 8-month-old infant presenting with severe nephrotic syndrome associated with heterozygous variants in both *LMX1B* and *TTC21B*, with spontaneous complete remission achieved by 12 months of age.

## Case presentation

An 8-month-old male infant born to non-consanguineous parents was admitted for bilateral eyelid swelling evolving over 10 days. 

The pregnancy had been complicated by maternal cytomegalovirus (CMV) infection during the first trimester. However, amniotic fluid PCR performed at five months of gestation and neonatal urine PCR testing were both negative. No other significant prenatal or perinatal events were reported. Family history revealed childhood nephrotic syndrome in the maternal grandfather, although detailed medical records were unavailable.

On physical examination, the patient presented with marked facial edema and bilateral pitting edema of the lower limbs. His weight was 8.32 kg (10th percentile), height 69 cm (10th percentile), and blood pressure 108/64 mmHg (>95th percentile for age). No dysmorphic features, nail abnormalities, skeletal anomalies, or other extrarenal manifestations suggestive of nail-patella syndrome were identified.

Laboratory findings are summarized in Table 1. Investigations revealed nephrotic-range proteinuria with a urinary protein-to-creatinine ratio of 25 g/g, severe hypoalbuminemia (17 g/L), hypercholesterolemia (total cholesterol 387 mg/dL), and preserved kidney function (serum creatinine 0.2 mg/dL). Additional findings included secondary hypothyroidism, low antithrombin III levels, and vitamin D deficiency. Complement levels were normal. No evidence of systemic disease was identified. Renal ultrasonography demonstrated kidneys of normal size, with preserved corticomedullary differentiation.

**Table 1:** Blood and urine test results on admission TSH: thyroid stimulating hormone; free T4: free thyroxine; 25-OH-vitamine D: 25-hydroxyvitamine D

	Results on admission	Results at 12 months of age	Normal values
Serum albumin	17 g/L	39 g/L	30–60 g/L
Total cholesterol	334 mg/dL	140 mg/dL	<170 mg/dL
TSH	5.75 mlU/L	2.07 mlU/L	0.3–4 mIU/L
Free T4	5.1 pmol/L	16.1 pmol/L	8-22 pmol/L
Fibrinogen	474 mg/dL	148 mg/dL	150–350 mg/dL
25-OH-vitamine D	<0.6 µg/L	40.8 µg/L	30–60 µg/L
Serum creatinine	>0.2 mg/dL	0.2 mg/dL	0.3–0.7 mg/dL
Proteinuria	25 g/g creatinine	0.42 g/g	<0.2 g/g
Microalbuminuria	21340 mg/g creatinine	10.2 mg/g creatinine	<30 mg/g

Because of the early onset of nephrotic syndrome and the high likelihood of an underlying genetic cause, genetic testing was performed, and corticosteroid therapy was withheld. Supportive treatment was initiated, including daily human albumin infusions administered through a central venous catheter (1-2 g/kg/day), anticoagulation prophylaxis, thyroid hormone replacement, vitamin D supplementation, and nutritional support (high-calorie enteral nutrition via a nasogastric tube).

Genetic findings

Genetic testing was performed using a targeted nephrotic syndrome gene panel with KAPA HyperCap v3 target enrichment (Roche Sequencing Solutions, Pleasanton, CA, USA) followed by next-generation sequencing on an Illumina platform (Illumina Inc., San Diego, CA, USA). According to the diagnostic laboratory report, targeted exons and exon-intron boundaries were covered with a mean sequencing depth of approximately 100× and a minimum coverage of 50× per base. Reported analytical sensitivity and specificity exceeded 99% for single-nucleotide variants and small insertions/deletions.

Analysis identified two heterozygous variants classified as variants of uncertain significance (American College of Medical Genetics and Genomics (ACMG) class 3 [10]):

*LMX1B*: c.1051G>A, p.(Gly351Arg) (NM_001174147.2)

*TTC21B*: c.2777G>A, p.(Arg926Gln) (NM_024753.5)

The *LMX1B* variant had not previously been reported in the literature. According to the diagnostic laboratory report, available in silico tools provided discordant predictions regarding pathogenicity, and splice prediction software suggested a possible effect on RNA splicing. However, no detailed prediction scores, RNA studies, or functional validation experiments were available. The affected amino acid residue is poorly conserved across species, which does not support a strong deleterious effect of the substitution. 

The *TTC21B* p.(Arg926Gln) variant is extremely rare in population databases and has been proposed as a variant of uncertain significance. Most available in silico prediction tools suggested a benign effect, and no functional studies have demonstrated pathogenicity.

Segregation analysis identified both variants in the patient's mother. She had no history of nephrotic syndrome and, at the time of evaluation, showed normal renal function (serum creatinine 0.64 mg/dL), normal urinary albumin excretion (urine albumin-to-creatinine ratio <30 mg/g), and no hypertension. The maternal grandfather carried only the LMX1B variant. According to family history provided by the patient's mother, he reportedly experienced frequently relapsing childhood nephrotic syndrome that eventually remitted. He died in 2024 from a brain tumor. No medical records were available to confirm the diagnosis, histological findings, treatment response, or long-term renal outcome.

Clinical outcome 

The patient's edema progressively improved under supportive treatment alone. Human albumin infusions were gradually discontinued as serum albumin levels increased and proteinuria decreased.

Complete clinical and biological remission was achieved at 12 months of age, with normalization of serum albumin levels and resolution of proteinuria. Blood pressure also normalized during follow-up. At the most recent evaluation, performed at three years and eight months of age, the child remained in complete remission without relapse. Kidney function, serum albumin levels, and urinary albumin excretion remained normal.

## Discussion

Infantile nephrotic syndrome is strongly associated with an underlying genetic cause, with pathogenic variants identified in approximately 70% of cases presenting during the first year of life [2]. Most genetically determined forms are characterized by persistent proteinuria, poor responsiveness to immunosuppressive therapy, and an increased risk of progression to chronic kidney disease [2]. The present case is therefore remarkable because of the complete and sustained spontaneous remission observed without corticosteroid or other immunosuppressive treatment.

Genetic analysis identified two heterozygous variants of uncertain significance in *LMX1B* and *TTC21B*, two genes previously implicated in hereditary kidney disorders. However, neither variant currently fulfills criteria for pathogenic or likely pathogenic classification, and the available evidence is insufficient to establish a definitive molecular diagnosis.

The *LMX1B* gene encodes a LIM-homeodomain transcription factor that plays a critical role in podocyte differentiation and glomerular basement membrane development. Pathogenic variants are classically associated with nail-patella syndrome but may also cause isolated renal disease without extrarenal manifestations [5,6]. The identified *LMX1B*variant, c.1051G>A p.(Gly351Arg), has not been previously reported. According to the diagnostic laboratory report, available prediction tools yielded discordant results regarding pathogenicity, while splice prediction software suggested a possible effect on RNA splicing. However, no detailed prediction scores, RNA studies, minigene assays, or other functional analyses were available. Consequently, any impact on RNA splicing remains hypothetical. Furthermore, the affected glycine residue is poorly conserved across species, suggesting limited evolutionary constraint and weakening the evidence supporting a deleterious effect of the amino acid substitution.

The *TTC21B* gene encodes intraflagellar transport protein 139, an essential component of retrograde ciliary transport. Biallelic pathogenic variants are associated with a spectrum of ciliopathies and glomerular diseases [7]. The identified *TTC21B* variant, p.(Arg926Gln), is extremely rare in population databases but remains classified as a variant of uncertain significance. Most available prediction tools suggest a benign effect, and no functional studies have demonstrated pathogenicity. Although heterozygous *TTC21B* variants have been proposed as modifiers of disease severity in selected renal disorders [11], their contribution to isolated infantile nephrotic syndrome remains uncertain.

A potential oligogenic contribution involving both variants was initially considered. Oligogenic inheritance has been proposed in selected forms of nephrotic syndrome and other inherited kidney diseases, where multiple variants may collectively influence disease expression [9]. In such models, one variant may act as a primary susceptibility factor while another modifies penetrance, severity, age at onset, or clinical outcome. However, evidence supporting oligogenic inheritance in pediatric nephrotic syndrome remains limited, and most reported cases involve other gene combinations.

Importantly, the familial segregation pattern observed in the present case does not support a simple oligogenic model. The patient's mother carries the same double-heterozygous genotype but has normal renal function, normal urinary albumin excretion, and no hypertension. This observation indicates that the coexistence of both variants is not sufficient by itself to cause disease. Furthermore, the maternal grandfather reportedly experienced childhood nephrotic syndrome while carrying only the *LMX1B* variant. Although this history is based solely on family reports and could not be independently verified, it further complicates the interpretation of the genetic findings. Taken together, these observations suggest incomplete penetrance, variable expressivity, the influence of additional genetic or environmental factors, or the possibility that one or both variants are incidental findings unrelated to the clinical presentation.

An alternative explanation should therefore be considered. The spontaneous and sustained remission observed in our patient is atypical for most monogenic forms of nephrotic syndrome and raises the possibility that the disease represented a transient or self-limited glomerular disorder occurring in a child who incidentally carries two variants of uncertain significance. Congenital cytomegalovirus infection was excluded, and no alternative secondary cause was identified during the diagnostic evaluation. Nevertheless, a transient environmental or infectious trigger cannot be completely excluded. Under this hypothesis, the identified variants may reflect genetic background variation rather than direct contributors to disease expression. 

Although most monogenic forms of nephrotic syndrome are associated with persistent proteinuria and progression to chronic kidney disease, substantial phenotypic variability has been reported. In particular, some patients with *NPHS1*-related disease have demonstrated unexpectedly mild clinical courses, including spontaneous partial or complete remission, illustrating that genotype-phenotype correlations are not always straightforward [2]. However, comparable reports involving *LMX1B* or *TTC21B* variants remain scarce. Consequently, the favorable clinical course observed in this patient should be regarded as unusual and not as a predictable consequence of the identified genetic findings.

One of the principal strengths of this case is the early use of genetic testing, which supported a conservative therapeutic approach and avoided unnecessary exposure to corticosteroids. Nevertheless, the case also illustrates the limitations of genetic testing when variants of uncertain significance are identified. Such findings may suggest possible biological pathways but cannot establish causality without supportive segregation data, functional studies, or independent replication in additional cases.

Long-term follow-up remains essential. Although the patient remains in complete remission at three years and eight months of age, the long-term prognosis cannot be predicted with certainty. The patient's mother, who carries the same double-heterozygous genotype, currently shows no evidence of renal disease. However, this observation should be interpreted cautiously, as age-dependent penetrance and variable expressivity have been described in several hereditary nephropathies [2,12,13]. Therefore, neither the pathogenic contribution of the identified variants nor their prognostic significance can be established based on the available segregation data alone.

## Conclusions

This case describes severe infantile nephrotic syndrome with sustained spontaneous remission in a child carrying two variants of uncertain significance in *LMX1B* and *TTC21B*. Although a genetic contribution cannot be excluded, the available evidence is insufficient to establish a causal oligogenic mechanism. The familial segregation pattern, including an unaffected mother carrying both variants, highlights the challenges of interpreting variants of uncertain significance in clinical practice. 

The unusual spontaneous remission observed in this patient expands the spectrum of outcomes reported in infantile nephrotic syndrome and emphasizes the importance of cautious genotype-phenotype interpretation. Early genetic testing remains valuable for therapeutic decision-making, but long-term clinical follow-up is required regardless of molecular findings.

## References

[1] Trautmann A, Boyer O, Hodson E (2023). IPNA clinical practice recommendations for the diagnosis and management of children with steroid-sensitive nephrotic syndrome. Pediatr Nephrol.

[2] Boyer O, Schaefer F, Haffner D (2021). Management of congenital nephrotic syndrome: consensus recommendations of the ERKNet-ESPN Working Group. Nat Rev Nephrol.

[3] Sadowski CE, Lovric S, Ashraf S (2015). A single-gene cause in 29.5% of cases of steroid-resistant nephrotic syndrome. J Am Soc Nephrol.

[4] Warejko JK, Tan W, Daga A (2018). Whole exome sequencing of patients with steroid-resistant nephrotic syndrome. Clin J Am Soc Nephrol.

[5] Harita Y, Kitanaka S, Isojima T, Ashida A, Hattori M (2017). Spectrum of LMX1B mutations: from nail-patella syndrome to isolated nephropathy. Pediatr Nephrol.

[6] Witzgall R (2008). How are podocytes affected in nail-patella syndrome?. Pediatr Nephrol.

[7] Reiter JF, Leroux MR (2017). Genes and molecular pathways underpinning ciliopathies. Nat Rev Mol Cell Biol.

[8] Davis EE, Zhang Q, Liu Q (2011). TTC21B contributes both causal and modifying alleles across the ciliopathy spectrum. Nat Genet.

[9] Crawford BD, Gillies CE, Robertson CC, Kretzler M, Otto EA, Vega-Warner V, Sampson MG (2017). Erratum to: evaluating Mendelian nephrotic syndrome genes for evidence for risk alleles or oligogenicity that explain heritability. Pediatr Nephrol.

[10] Richards S, Aziz N, Bale S (2015). Standards and guidelines for the interpretation of sequence variants: a joint consensus recommendation of the American College of Medical Genetics and Genomics and the Association for Molecular Pathology. Genet Med.

[11] Huynh Cong E, Bizet AA, Boyer O (2014). A homozygous missense mutation in the ciliary gene TTC21B causes familial FSGS. J Am Soc Nephrol.

[12] Wang D, Chen X, Wen Q, Li Z, Chen W, Chen W, Wang X (2022). A single heterozygous nonsense mutation in the TTC21B gene causes adult-onset nephronophthisis 12: a case report and review of literature. Mol Genet Genomic Med.

[13] Hildebrandt F (2010). Genetic kidney diseases. Lancet.

